# Global budget versus cost ceiling: a natural experiment in hospital payment reform in the Netherlands

**DOI:** 10.1007/s10198-019-01114-6

**Published:** 2019-09-16

**Authors:** Katalin Gaspar, France Portrait, Eric van der Hijden, Xander Koolman

**Affiliations:** 1 Department of Health Sciences, Health Economics Section, Talma Institute/VU University Amsterdam, De Boelelaan 1085, 1081 HV Amsterdam, The Netherlands; 2 Zilveren Kruis (Achmea), Amersfoort, The Netherlands

**Keywords:** Provider payment, Global budget, Provider incentive, Policy evaluation, Regulated competition, The Netherlands, I11, I13, I18

## Abstract

Global budget (GB) arrangements have become a popular method worldwide to control the rise in healthcare expenditures. By guaranteeing hospital funding, payers hope to eliminate the drive for increased production, and incentivize providers to deliver more efficient care and lower utilization. We evaluated the introduction of GB contracts by certain large insurers in Dutch hospital care in 2012 and compared health care utilization to those insurers who continued with more traditional production-based contracts, i.e., cost ceiling (CC) contracts. We used the share of GB hospital funding per postal code region to study the effect of contract types. Our findings show that having higher share of GB financing was associated with lower growth in treatment intensity, but it was also associated with higher growth in the probability of having at least one hospital visit. While the former finding is in line with our expectation, the latter is not and suggests that hospital visits may take longer to respond to contract incentives. Our study covers the years of 2010–2013 (2 years before and 2 years following the introduction of the new contracts). Therefore, our results capture only short-term effects.

**Electronic supplementary material:**

The online version of this article (10.1007/s10198-019-01114-6) contains supplementary material, which is available to authorized users.

## Introduction

Global budget (GB) arrangements have long been considered as the magic bullet to reach more efficiency in healthcare [1]. In the United States, discussion regarding their implementation dates back as far as the Health Security Act of 1993 during the Clinton administration [2]. Although rejected in the United States then, the idea has become a key element in payment reforms around the world (e.g. France—[3]; Taiwan—[4–6]; Germany—[7]. In 2010, it became one of the central pillars of Medicare’s Alternative Payment Reform and, as a pilot study, implemented in the state of Maryland.

A GB arrangement entails giving a set of providers a pre-specified budget to finance all costs associated with the care of a group of patients for a fixed period of time (usually 1 year) [8]. Providers’ funding is not only capped, but also is guaranteed with certain restrictions on quality and accessibility of care, in the hope that.

“… providing fixed, predictable revenue allows hospitals to focus on value rather than volume and rewards them for investing in population health improvement” [9]

Global budgeting was initiated in the Netherlands in 2012, when certain large health insurers introduced hospital GBs as their primary payment method of hospital care. This arrangement is closest to that used in Maryland: budgets are allocated to hospitals and are enforced using ex post price adjustments. But in contrast to other countries, in the Netherlands not all insurers introduced GBs, which resulted in two different payment methods used in parallel: a production-based funding with a cap on expenditure and guaranteed GB funding.

Due to the small size of the country, large Dutch health insurers generally have contracts with all major hospitals and hospitals treat patients from several insurers. Therefore, a typical hospital ended up with a mixture of funding sources: a certain fraction of the total hospital funding was guaranteed, while the rest came from production-based sources. The exact share of the two sources depended on the market share of health insurers in that region. Since insurers followed the same contracting strategy in nearly all hospitals and this strategy was exogenously determined from the characteristics of the individual hospital, in this paper we argue that hospitals were randomly assigned different degrees of production incentives in the form of contracts, depending on the market share of insurers in the hospital. This resulted in a natural experiment and an ideal ground to evaluate the effect of contract incentives on production.^1^

The aim of our research is to quantify the effect of guaranteed funding by GB contracts on hospital utilization. There is a large body of literature investigating how the elimination of production incentives leads to lower spending. The most prominent example is the transition from a fee-for-service (FFS) system to a prospective payment system in the United States, where providers responded by large drop in output when the marginal reimbursement of additional services went from positive to zero [10–12]. Our paper analyzed the probability of having a hospital visit and the intensity of treatment per patient as a result of a similar shift in payment systems. Similarly to their findings, our initial expectation was a negative correlation between health care utilization and the share of GB financing.

Our research uses claims-level data provided by the second largest health insurer in the Netherlands (Coöperatie VGZ), with approximately 20% of the national market share. We build on the assumption that providers in the Netherlands treat patients independent of which private health insurer they are insured with. This assumption, albeit to our knowledge unproven, seems reasonable given the unique structure of Dutch health care system: nearly all residents of the Netherlands are insured by one of the several private health insurers [13] and practically all hospital care (excluding only a short list of treatments, e.g., certain plastic surgery and fertility treatments) are included in the basic package provided by all health insurers. Therefore, we consider our findings using one insurer indicative of the national trends.

Prior empirical findings on global budgeting are not yet conclusive and results vary with the type of arrangement that is being studied. One strand of literature focusing on systems with ex post price adjustment and regional- or sectoral-level budget allocation has found an increase in healthcare utilization as a result of the policy. This unanticipated result was found in two provinces in Canada [14], in German ambulatory care [7] and most recently in hospital care in Taiwan [4]. Authors in all three cases argue that GB payment with price adjustment and with FFS providers is a form of common‐pool resources, where, instead of cooperating to keep volumes low and thereby prices high, each physician attempts to individually maximize revenues leading to high volumes and low prices. This finding is contrasted in a recent paper on the arrangement in Maryland, where budgets were allocated to hospitals with ex post price adjustments, and found no significant change in hospital utilization [15]. Alternatively, the providers’ budget is simply capped and earmarked to the hospital without the use of shadow prices for underlying services, and hence no need for price adjustments. Such arrangements have been shown to successful curb spending and volumes in France [3] and in Ontario [16].

### Institutional setting

In 2006, the Netherlands embarked on a major transformation of its healthcare system from a centrally regulated scheme towards one that is based on the principles of managed competition. The reform was implemented in phases: each year a pre-defined part of hospital services was transferred over to the competition segment, where prices and volume were freely negotiated between health insurers and hospitals. Hospitals enjoyed predominantly open-ended funding in this segment of care. However, as hospital care was gradually liberalized, healthcare expenditure and utilization began to grow exponentially (rising from 10.7 to 11.9% of the GDP from 2006 to 2011) [17]. This trend and the general economic downturn set in motion a new wave of reforms and a general agreement between the government, health insurers and providers to bring healthcare spending under control. The new general agreement stipulated that expenditure growth must be below 2.5% (later 1.0%) per year for the period of 2012–2015 [13]. Providers became risk-bearing for all costs incurred beyond this level and were also hit with the elimination of retrospective risk equalization for case-mix differences between insurers in the same year [18, 19]. Insurers responded to the increase in risk burden by re-negotiating contracts with hospitals to include expenditure caps [13].

#### Hospital financing

Nearly, all hospital expenditure became capped in 2012, but insurers followed different strategies in designing the details of the new contracts. Two types of contracts became prevalent: one based on hospital production but with an overall cap on expenditures and pre-negotiated prices [cost ceiling (CC) contract]; the other with guaranteed budget independent of production (GB contracts). Although both types are budgeted contracts (i.e., both include a cap on the amount of expenditure allowed), there is a distinct difference in incentives: while funding must be ‘earned’ (i.e., services must be provided) with the former, funding amount is guaranteed for the hospital with the latter, which means hospitals are under less production pressure.

The insurance market in the Netherlands consists of four major health insurers with nationwide networks and a few smaller players with more limited geographical coverage. The four main insurers (Zilveren Kruis, VGZ, CZ and Menzis) together represent 89.8% of the total health insurance market [20]. Major insurers tend to have contracts with each general hospital and university medical center for all services included in the basic insurance package and potentially more. In 2012, insurers opted to follow one of the two contracting strategies described above. In the majority of the cases, they followed the same strategy in all of their contracts nationwide, which, due to the insurers´ varying market share, resulted in strong regional variation in contract types.

#### Physician payments

Physicians are either self-employed or paid by a monthly salary. Self-employed physicians’ income is mainly determined by their production, while salaried specialists’ remuneration is independent of the amount of care provided. This distinction in payment types may affect our results, since the financial incentives of the physicians and that of the hospital may be misaligned. While hospital management with large share of GB funding is incentivized to limit production, self-employed physicians may choose to maintain utilization at a high level to maximize their own profits. This will dampen the effects GBs have on production. The exact share of self-employed to salaried physicians per hospital is difficult to establish for our research period. Kroneman et al. have reported that UMC staff is generally paid by salary [13]. In addition, Douven et al. reported that in 2019, majority of physicians (68%) were self-employed, but that there was a relatively large group (approx. 15% of treatments in their data) where remuneration type was unknown and were suspected to be self-employed physicians working in small private clinics [21]. These publications lead us to believe that majority of GH specialists were, at the time, paid as self-employed and UMC staff were paid on salary. For this reason, and due to differences in funding, we explicitly control for treatment performed in UMCs.

#### Concurrent policy changes

**Fig. 1:**
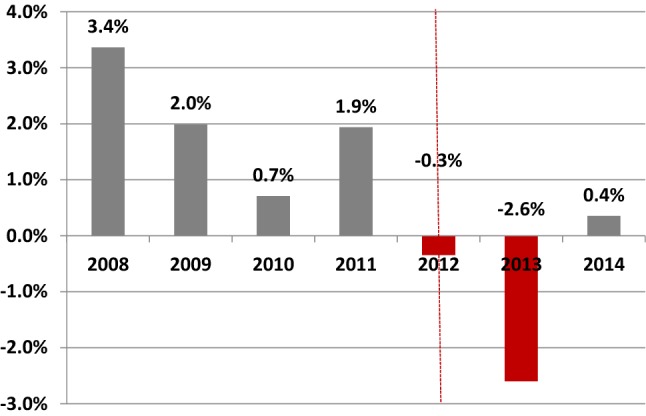
Change in the number of patients treated in hospital care, national figures for all health insurers. Data publicly available per municipality. It has been aggregated to obtain national figures. Vektis Zorgprisma Publiek, Available: https://www.zorgprismapubliek.nl/producten/ziekenhuiszorg/ontwikkeling-medische-specialistische-zorg/ontwikkeling-aantal-patienten/

**Fig. 2:**
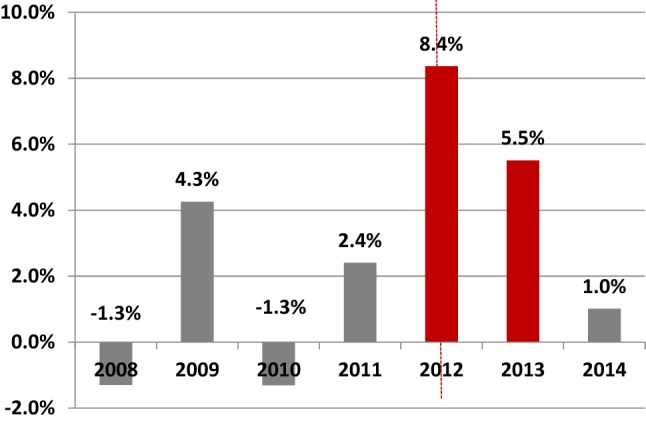
Change in average hospital costs per patient per year, national figures for all health insurers. Data publicly available per municipality. It has been aggregated to obtain national figures. Vektis Zorgprisma Publiek, Available: https://www.zorgprismapubliek.nl/producten/ziekenhuiszorg/ontwikkeling-medische-specialistische-zorg/ontwikkeling-aantal-patienten/

A number of additional policy changes occurred during the period of our analysis. The DRG system transitioned from a system covering a combination of disease-treatment groups to one mainly focusing on disease. As a part of this transition, the number of available products went from nearly 30 thousand products up until the year 2011 to just 4400 in 2012 [22]. Front-end deductibles were increased in 2012 and once again in 2013 (2011: €170; 2012: €220; 2013: €350). The individual effects of these policy changes on overall hospital spending and patient volumes have not been scientifically investigated. However, according to national figures, there was a drop in patient volumes (the number of patients treated in hospital care dropped by 0.3% in 2012 and by 2.6% in 2013 shown in Fig. 1) and a pronounced increase in the average hospital cost per patient (8.4% in 2012 and 5.5% in 2013 shown in Fig. 2).

## Data and methods

### Data

Our dataset includes claims data provided by Coöperatie VGZ and hospital contract information provided by the National Healthcare Authority of the Netherlands (NZa). The claims dataset comprises all hospital care registered for the period 2010 and 2013, including inpatient and outpatient care for all policy-holders excluding wholesale clients, and except physiotherapy, particular high-priced medications and certain diagnostics required by the general practitioner, which are excluded from the DRG system. During the period of our analysis, the maximum amount of time a claim could be open was 1 year; after 1 year, claims were automatically closed and processed.

Hospital contracts were collected for the years 2012–2013 by the NZa. Our team then used these contracts to extract and code contract details for a total of 80 hospitals [72 general hospitals and 8 university medical centers (UMCs)] and the four main health insurers in the Netherlands. The following contract information was recorded:

- type of contract (CC, GB, or other)
- budget amount per contract

The GB share of the total hospital budget was calculated according to formula (1), where the total budget amount is restricted to the four main insurers. This variable was used to indicate contract type in our analysis.

1 $${\text{Contract type = }}\frac{\text{GB amount}}{\text{Total budget amount}}$$

Contract-type information was connected with claims data provided by Coöperatie VGZ based on official hospital registration codes (Algemeen GegevensBeheer-codes or AGB-codes). Around 82% of the claims in our dataset had contract information attached. Claims without contract information were excluded from the analysis. To allow for a 2012 change in DRG product definitions, hospital spending was aggregated to the level of the patient.

Coöperatie VGZ had only CC contracts during the period of our study with a strong base in the North-Brabant region. However, as the second largest insurer, it has contractual relationships with all major hospitals in the Netherlands.

### Outcome variables

#### Analysis 1: probability of hospital visits

The probability of having a hospital visit was investigated using the binary variable ‘Treat’ that is equal to 1 when an individual policy-holder had a claim that year and equal to zero otherwise (denoted by *T*_it_ for individual i in year t).

#### Analysis 2: treatment intensity

Treatment intensity was proxied by total hospital spending per patient per year using national prices (denoted by $\overline{\text{HCE}}_{\text{it}}$ for individual *i* and year *t*). We used national average prices per product per year to average out price differences between hospitals to obtain true differences in treatment intensity.

### Regional contract-types variables

Instead of allocating not treated individuals to ‘potential’ hospitals, we calculated the average contract type for region r in year *t* (${\text{RCT}}_{\text{rt}}$) based on the claims registered in the region and used this variable to control for contract type. Four-digit postal codes served as the administrative units for regions. Since GB funding was introduced in 2012, RCT_rt_ is equal to zero before 2012. In some smaller postal codes, the number of claims registered was low, which could bias our RCT_rt_ calculation. To avoid this bias, we excluded regions with less than 100 claims per postal code per year.

### Share of treatments performed at University Medical Centers (Share_UMC)

Share_UMC_it_ is a continuous variable indicating the share of services an individual *i* has received in a UMC in year *t* (based on the number of claims). It is equal to 1 if all claims of an individual in a certain year were registered in a UMC and equal to zero if all claims were registered in GH.

### Additional independent variables

The time period was indicated by the variable Post_*i*_, where the years 2012 and 2013 were equal to 1. Years of age was categorized into 19 groups by 5-year increments, beginning with 0–5 years. Sex was added to the regression as a binary variable equal to 1 if female. SES index was used to control for socio-economic differences between regions. The SES index was calculated by the social and cultural planning office (SCP) for the year 2013 at the four-digit postal code level based on average income, poverty, level of education and employment figures (See Table 1 for list of variables).

**Table 1:** List of variables

Name/abbreviation	Variable definition
$T_{\text{it}}$	Received hospital treatment in a given year, =1 if yes, 0 otherwise
${\text{Post}}_{\text{i}}$	=1 for years 2012–2013, 0 otherwise
$\overline{{{\text{HCE}}_{\text{it}} }}$	Hospital related healthcare expenditure at national average prices, proxy for Treatment intensity
${\text{RCT}}_{\text{rt}}$	Average contract type per postal code per year
${\text{Share}}\_{\text{UMC}}_{\text{it}}$	Share of claims registered in UMC per patient per year
${\text{Age }}0\_4 - {\text{Age }}85 +$	Age categorized into 19 groups by 5-year increments
${\text{Female}}_{\text{i}}$	Female = 1 if female, 0 otherwise
${\text{SES}}_{i}$	Socio-Economic Status index

## Methods

### Analysis 1: probability of hospital visits

In total, five models (three logit and two conditional logit) were estimated to statistically test the effects of contract type on the probability of having a hospital visit. Conditional logit models were used in addition to the conventionally used logit models to better control for time-invariant individual effects. In Models 1 and 4, the average trend was demonstrated before adding contract types. In Model 2, contract types were added to the regression but without additional controls. Models 3 and 5 represent the complete model after controlling for contract type and individual characteristics (in Model 3 by explicitly controlling for case-mix variables and in Model 5 by utilizing a conditional logit method).

For logit models:

2 $${\text{Prob}}({T_{\rm it}} = 1|{Z_{\rm it}}) = \frac{{{{\text{e}}^{\left({\alpha + \beta {\text{Pos}}{{\text{t}}_i} + \gamma \left({{\text{Pos}}{{\text{t}}_i}*{{\mathop {RCT}\limits}_{\text{rt}}}} \right) + \theta {Z_{\text{it}}} + {e_{\text{it}}}} \right)}}}}{{1 + {{\text{e}}^{\left({\alpha + \beta {\text{Pos}}{{\text{t}}_i} + \gamma \left({{\text{Pos}}{{\text{t}}_i}*{{\mathop {RCT}\limits}_{\text{rt}}}} \right) + \theta {Z_{\text{it}}} + {e_{\text{it}}}} \right)}}}}$$

For conditional logit models:

3 $${\text{Prob}}({T_{\text{it}}} = 1|{X_i}) = \frac{{{{\text{e}}^{\left({{\alpha_i} + \beta {\text{Pos}}{{\text{t}}_i} + \gamma \left({{\text{Pos}}{{\text{t}}_i}*{{\mathop {\text{RCT}}\limits}_{\text{rt}}}} \right) + {e_{\text{it}}}} \right)}}}}{{1 + {{\text{e}}^{\left({{\alpha_i} + \beta {\text{Pos}}{{\text{t}}_i} + \gamma \left({{\text{Pos}}{{\text{t}}_i}*{{\mathop {\text{RCT}}\limits}_{\text{rt}}}} \right) + {e_{\text{it}}}} \right)}}}}$$

We used individual policy-holders as the unit of the analysis who either received some kind of treatment ($T_{\text{it}}$ = 1) or did not ($T_{\text{it}}$ = 0). ${\text{Post}}_{i}$ indicates the period of the analysis: ${\text{Post}}_{i} = 1$ for the years 2012–2013, and 0 otherwise. ${\text{RCT}}_{\text{rt}}$ denotes the average contract type per region calculated according to Eq. 1. $Z_{\text{it}}$ denotes specific case-mix variables (age groups, sex and SES index per postal code). $X_{i}$ is a collection of all time-constant individual characteristics.

An overwhelming majority of papers on patients’ hospital choice have shown that travel time is the most important factor in patients’ decision on which hospital to visit, and that the effect of all other patients and hospital characteristics is negligible [23]. The decision to visit a hospital by a patient, and the decision to see a patient by a doctor are binary (visit vs. not visit) and do not depend on hospital type. Therefore, in the above analysis, we do not differentiate between visits to a GH or UMC, as hospital type is irrelevant for the decision of the patient.

#### Hypothesis 1

Growth in probability of receiving treatment is lower for regions where the share of GB funding is higher. That is $\gamma$ is < 0.

### Analysis 2: treatment intensity

In total, seven models (four pooled difference-in-difference regressions and three individual fixed effects) regressions were performed to evaluate the significance of contract types (${\text{RCT}}_{\text{rt }}$) on treatment intensity. ${\text{RCT}}_{\text{rt}}$ is interacted with our variable indicating the share of claims registered at UMCs (${\text{Share}}\_{\text{UMC}}_{it}$) to test any deviations from the general trend by UMC patients. Our dependent variable is hospital spending per patient using average national prices ($\overline{{{\text{HCE}}_{\text{it}} }}$). The parameter $\alpha_{i}$ is a constant intercept ($\alpha$) in Models 1–4 and denotes individual fixed effects in Models 5–7.

The specification of the regressions is as follows:

4 $$\begin{array}{ll} \log \left( {\overline{\text{HCE}}_{\rm it}} \right) & = \alpha_{i} + \beta_{1} {\text{Post}_{i}} + \beta_{2} {\text{RCT}_{\rm rt}} + \beta_{3} {\text{Share}} \_{{\rm UMC}_{\rm it}} + \beta_{4} {\text{Post}_{i}} *{\text{RCT}_{\rm rt}} + \beta_{5} {\text{Post}_{i}} *{\text{Share}} \_{{\rm UMC}_{\rm it}} \\ & \quad + \beta_{4} {\text{Post}_{i}} *{\text{Share}} \_{{\rm UMC}_{\rm it}} * {\text{RCT}}_{\rm rt} + \theta X_{\rm it} + e_{\rm it} \\ \end{array}$$

This analysis is restricted to policy-holders who received treatment during the year (i.e., $\overline{{{\text{HCE}}_{\text{it}} }}$ > 0).

As with most health care cost data, our dependent variable ($\overline{{{\text{HCE}}_{\text{it}} }}$) is log-normally distributed and skewed to the right, which may lead to biases in linear regressions. We use log-transformation to assure the unbiasedness of our results.

#### Hypothesis 2

Growth in treatment intensity is lower for regions where the share of GB funding is higher. That is, *β*_4_ is < 0.

## Results

### Descriptive statistics

Table 2 presents the descriptive statistics. Our final dataset includes 13.6 million individuals, (or 3.4–3.5 individuals per year). 5.6 million individuals (or 1.4 million individuals per year) have some kind of hospital treatment registered during this period. The share of treated individuals followed a declining trend from 40.9% in 2011 to 39.6% in 2013, with a 1.5 and 1.3% decline in 2012 and 2013. Hospital spending per year for treated individuals rose at steady rate of 1.7% in years 2011 and 2012, followed by a 5.1% in 2013.

**Table 2:** Dependent variable: treat

	2010 (*n *= 3429838)	2011 (*n *= 3396123)	2012 (*n *= 3444890)	2013 (*n *= 3367857)	Overall (*n *= 13638708)
% of policy-holders treated					
Mean (SD)	40.8 (49.1)	41.0 (49.2)	40.5 (49.1)	39.8 (49.0)	40.5 (49.1)
Median [min, max]	0.0 [0.0, 100.0]	0.0 [0.0, 100.0]	0.0 [0.0, 100.0]	0.0 [0.0, 100.0]	0.0 [0.0, 100.0]
HCE per policy-holder (€—National prices)					
Mean (SD)	932 (3560)	952 (3612)	953 (3660)	987 (4086)	956 (3734)
Median [Min, Max]	0 [0, 281544]	0 [0, 269002]	0 [0, 343690]	0 [0, 486065]	0 [0, 486065]
HCE per patient (€—National prices)					
Mean (SD)	2287 (5290)	2323 (5352)	2359 (5463)	2481 (6183)	2361 (5578)
Median [min, max]	683 [40, 281544]	689 [43, 269002]	759 [78, 343690]	786 [71, 486065]	727 [40, 486065]
Average contract type by postal code (%)					
Mean (SD)	0.0 (0.0)	0.0 (0.0)	30.0 (23.0)	34.7 (0.23.6)	16.1 (23.1)
Median [min, max]	0.0 [0.0, 0.0]	0.0 [0.0, 0.0]	22.0 [1, 81.0]	27.1 [0.0, 80.8]	0.0 [0.0, 81.3]
Age of policy-holders (years)					
Mean (SD)	41.0 (22.9)	41.3 (23.0)	41.8 (23.1)	42.3 (23.2)	41.6 (23.1)
Median [min, max]	42.0 [0.0, 108.0]	42.0 [0.0, 109.0]	43.0 [0.0, 110.0]	44.0 [0.0, 111.0]	43.0 [0.0, 111.0]
Age of patients (years)					
Mean (SD)	47.4 (23.6)	47.8 (23.6)	48.7 (23.6)	49.4 (23. 6)	48.3 (23.6)
Median [min, max]	51.0 [0.0, 108.0]	51.0 [0.0, 109.0]	53.0 [0.0, 110.0]	54.0 [0.0, 111.0]	52.0 [0.0, 111.0]
Female (%)					
Mean (SD)	52.3 (49.9)	52.1 (50.0)	52.0 (50.0)	51. 9 (50.0)	52.1 (50.0)
Median [min, max]	100.0 [0.0, 100.0]	100.0 [0.0, 100.0]	100.0 [0.0, 100.0]	100.0 [0.0, 100.0]	100.0 [0.0, 100.0]
SES index					
Mean (SD)	− 0.04 (1.00)	− 0.023 (0.97)	− 0.02 (0.98)	− 0.03 (0.98)	− 0.03 (0.98)
Median [min, max]	0.05 [− 7.62, 2.83]	0.06 [− 7.62, 2.83]	0.07 [− 7.62, 2.83]	0.06 [− 7.62, 2.93]	0.06 [− 7.62, 2.93]
% of UMC treatment per patient with any UMC claims					
Mean (SD)	75.3 (31.0)	74.5 (31.2)	74.5 (31.3)	74.1 (31.5)	74.6 (31.3)
Median [min, max]	100.0 [1.4, 100.0]	100.0 [01.4, 100.0]	100.0 [1.4, 100.0]	100.0 [1.5, 100.0]	100.0 [1.4, 100.0]

Approximately, 30% of hospital spending originated from GB sources in 2012 in our dataset, this rose slightly to 35% in 2013 due to shift towards more GB funding in a small number of hospitals. Since the provider of our data has a stronger market position in areas where CC is the primary funding method, this average is likely underestimated. The variable RCT_rt_ has a strong regional distribution due to the regional market position of health insurers. Omitting postal codes due to small sample size of claims (*n *< 100 per year) led to the elimination of < 1% of our dataset.

The average of the continuous variable Share_UMC_it_ is equal to 75%. This means that 75% of claims originated from UMCs for patients with any UMC treatment, while the rest were registered at GHs. The average policy-holder in our dataset is slightly older than the national average (the percentage of policy-holders 65 and older in our dataset is 18.1% compared to the national average of 16.2%) [24].

### Analysis 1: probability of hospital visit

Table 3 presents the odds ratios for five models (3 logit and 2 conditional logit) performed on the probability of a hospital visit. (See Appendix Tables 1 and 2 for the complete regression outputs.) The general declining trend is demonstrated by the odds ratio for the variable Post_*i*_ in Model 1 and in Model 4 without any other control variables. The odds of a hospital visit were 0.968 before, and 0.975 after eliminating time-invariant individual characteristics (seen in Models 1 and 4, respectively). The association between the probability of receiving treatment and GB share is indicated by the odds ratio on the interaction term Post_i_ * RCT_rt_: 0.758 in Model 2 (without any correction), somewhat higher (0.862) in Model 3 (with correction for case-mix differences), but 1.034 in Model 5 after correction for time-invariant individual characteristics, indicating a positive relationship.^2^

**Table 3:** Analysis 1: probability of hospital visit—Odds ratios

	Logistic			Conditional logistic	
	(1)	(2)	(3)^a^	(4)	(5)
Dependent variable: treat					
Post	0.968***	1.058***	0.988***	0.975***	0.965***
	(1.001)	(1.002)	(1.002)	(1.001)	(1.002)
Post*RCT		0.758***	0.862***		1.034***
		(1.003)	(1.003)		(1.006)
Observations	13,638,708	13,638,708	13,638,708	13,638,708	13,638,708
Log Likelihood	− 9,205,509	− 9,202,064	− 8660,015	− 2522,906	− 2522,891

*p < 0.1

**p < 0.05

***p < 0.01

^a^Including case-mix variables (age groups, sex, SES index)

The dissimilarities in results suggest that although higher GB shares were associated with considerably lower odds of having a hospital visit, this negative relationship was entirely explained by differences in individual characteristics within regions. The odds rise once case-mix variables are added to the regression and a positive relationship surfaces once time-invariant differences are accounted for.

### Analysis 2: treatment intensity

In Table 4, we present the results for seven linear regressions on log($\overline{{{\text{HCE}}_{\text{it}} }}$) (see appendix Table 3 for the complete regression outputs). The large and positive coefficient on the Post_*i*_ and Post_i_ * Share_UMC_it_ variables throughout the seven models demonstrates the increase in overall hospital spending in the second half of the period. However, its magnitude seems to shrink once case-mix variables are added (Model 4), and shrink even further once time-invariant individual effects are eliminated (Models 5–7). The coefficient on the variable Share_UMC_it_ indicates substantially higher spending per patient per year at UMCs even at the base years of 2010 and 2011. This is to be expected, as UMCs generally treat more complex patients and incur higher costs. Then again, their growth in 2012–2013 is also larger than in GHs.

**Table 4:** Analysis 2: treatment intensity

	OLS				Panel Linear		
	(1)	(2)	(3)	(4)^a^	(5)	(6)	(7)
Dependent variable: log (HCE)							
Post	0.079***	0.113***	0.098***	0.065***	0.033***	0.043***	0.034***
	(0.001)	(0.002)	(0.002)	(0.002)	(0.001)	(0.002)	(0.002)
Post*RCT		− 0.110***	− 0.096***	− 0.042***		− 0.035***	− 0.015***
		(0.003)	(0.004)	(0.004)		(0.005)	(0.005)
Share_UMC			0.152***	0.192***			0.230***
			(0.003)	(0.003)			(0.005)
Post*Share_UMC			0.104***	0.079***			0.064***
			(0.005)	(0.005)			(0.006)
Post*RCT*Share_UMC			− 0.017	0.103***			− 0.157***
			(0.012)	(0.012)			(0.016)
Constant	6.714***	6.714***	6.698***	7.293***			
	(0.001)	(0.001)	(0.001)	(0.006)			
Observations	5523,626	5523,626	5523,626	5523,626	5523,626	5523,626	5523,626
*R*^2^	0.001	0.001	0.003	0.053	0.0003	0.0003	0.001
Adjusted *R*^2^	0.001	0.001	0.003	0.053	− 0.906	− 0.906	− 0.904

*p < 0.1

**p < 0.05

***p < 0.01

^a^Including case-mix variables (age groups, sex, SES index)

The coefficient on the Post_i_ * RCT_rt_ interaction term indicates a negative and significant correlation between treatment intensity and GB share (4–11% in Models 1–4). Even though its magnitude is considerably smaller in Models 5–7 (3.5 and 1.5% in Models 6 and 7) after individual case-mix differences are accounted for, it remains significant and negative throughout all models. The coefficient on the interaction term Post_i_ * Share_UMC_it_ * RCT_rt_ indicates a negative and significant relationship and unlike before, this relationship becomes stronger in absolute terms once individual differences are corrected for (− 1.3% in Model 3 to − 15.7% in Model 7).^3^ However, one must bear in mind that this is an interaction term between one binary and two continuous variables. The total term has a mean of 21.4% (± 20.9%) for individuals with any UMC claim and a maximum value of 81.3%. Therefore, in our dataset, the real effect is at maximum 12.7%.

Overall, our analysis demonstrates a negative and statistically significant correlation between the evolution of treatment intensity and the share of GB financing prevalent in the region. This relationship remains robust in all specifications of our model and after eliminating invariant individual differences.

## Discussion

The purpose of our study was to test whether and how financial incentives in contracts between hospitals and health insurers affected physicians’ decision to provide treatment. We evaluated the responses to two different financing arrangements that were introduced in 2012 in the Dutch hospital market: one based on fixed and guaranteed funding independent of production (GB contract), and a more traditional arrangement based on production with a cap on expenditure (CC contract). We hypothesized that by receiving guaranteed funding, care providers had an incentive to lower utilization and costs.

Since health insurers followed the same contracting strategy for the large majority of their contracts, and since large health insurers generally had contracts with all hospitals, otherwise comparable hospitals ended up with varying degrees of production incentives. This enabled us to estimate the average effect of CC compared to GB financing on the probability of a hospital visit and treatment intensities.

We tested our hypothesis using claims data provided by the second largest health insurer in the Netherlands. In practice, treating physicians usually did not know patients’ insurer. Therefore, we think it is safe to assume that physicians treated patients independent of where they were insured. Hence, our results may be indicative of the national trends. Our data encompass 4 years, 2 years before and 2 years after the introduction of the new contracts.

In line with the national trends, our results demonstrated a pronounced decline in the likelihood of hospital visits occurring in the second half of our study (2012–2013). However, the relationship between GB share and the probability was unclear, and the direction of the relationship was different depending on the specification of our model. Conditional on initial individual characteristics, our results indicated a positive correlation, which was in contrast to our initial expectation. One possible explanation could be linked to the decline in the intensity of care: an increase in physicians’ free capacity due to a drop in the intensity of treatments provided (see below) might have led to more patients being seen in the short-run (e.g., to eliminate already existing waiting lists), and that the effects of guaranteed budgets on the probability of a hospital visit may take more years to materialize.

On the other hand, treatment intensity, proxied by hospital spending per patient at national average prices, seemed to have a negative and statistically significant relationship with regional contract types. Treatment intensity has increased in the second half of the research period, but the growth is lower in regions where the share of GB financing in hospitals is larger. The direction of this relationship remained unchanged after controlling for case-mix differences and after eliminating time-invariant individual effects, but the magnitude diminishes in absolute terms from − 11% to − 1.5% for patients only treated in GHs and increases from − 1.6% to − 15.5% assuming all care of the individual is performed in UMCs. Hence, our findings are in line with our initial expectation that providers reduce or at least slow down their growth in intensity as a response to guaranteed budgets, although the effect seems to be strongest for patients treated by UMCs. This latter finding seems logical knowing that most physicians working in UMCs are paid on monthly salaries; therefore, their personal financial incentives are in line with that of the hospital when the hospital’s GB share is large.

Our paper builds on a rich dataset of claims-level data provided by one of the largest Dutch health insurers. However, having only one insurer in our dataset is also the main limitation of this paper as this may lead to unwarranted individual-level selection bias. Our dataset contains the most diverse pool of policy-holders where it has a strong market share. Policy-holders in other regions may somehow differ from the average policy-holders (e.g., they may be younger and/or more mobile than the average). Regional contract types also display a clear regional correlation as this is also primarily determined by the insurers market share. This may result in spurious correlation between GB shares and health care utilization and could lead to an inappropriate interpretation of our findings. We have utilized panel data analysis to circumvent these problems. Nonetheless, repeating our analysis on national data is advisable.

Furthermore, our research period contains data for only 2 years following the change in contracts, which may be inadequate timeframe to evaluate the full effect of global budgeting. The contracting strategy of insurers changed again in 2014, which prohibited us from extending our research to further years. Also, there were other concurrent policy changes that occurred in the year 2012, which were unrelated to hospital contract type. Overall, these changes led to a decrease in patient volumes (Fig. 1) and a considerable increase in hospital spending per patient (+8.5% in 2012) (Fig. 2), which indicates that hospital production was somehow affected by these policy changes. As funding must be ‘earned’ (i.e. services must be provided) with CC-funding, hospitals in this category are under more production pressure, especially those hospitals experiencing a decrease in patient volumes. Therefore, it follows that the incentive to increase production during the transitionary period was stronger for mainly CC financed hospitals than for GB financed hospitals with guaranteed funding. Hence, the policy changes of 2012 may have strengthened the difference in utilization by contract types in our findings.

Our paper has demonstrated that the introduction of GB contracts did not lead to lower growth in the probability of hospital visits for our cohort of individuals during the 2 years following the introduction of the new contracts, but that it led to lower growth in intensity of care in particular for patients treated at UMCs. Our findings are statistically and economically significant and may indicate similar trends nationally. When generalizing our findings one must bear in mind that they represent short-term results and that they may have been affected by the institutional setting of the time. Nonetheless, we consider our results strong, indicating that appropriate contract design may lead to lower health care utilization per patient and lower health care cost growth.

## Electronic supplementary material

Below is the link to the electronic supplementary material.

Supplementary material 1 (DOCX 23 kb)

Supplementary material 2 (DOCX 23 kb)

Supplementary material 3 (DOCX 27 kb)

## Footnotes

1 Natural experiment (or natural assignment) is a naturally occurring event that randomly assigns individuals into treated or control groups, mimicking a randomized controlled experiment (RCE). This technique can then be used to answer certain research questions in situations where RCE’s (e.g., in the case of most policy changes) are not feasible (Katikireddi et al. 2017).

2 Using conditional logit may introduce a small bias in our regression by limiting our dataset to individual with more than one observation, similarly to a balanced panel. We tested the magnitude of this bias by performing Models 1–3 in Table 3 using unbalanced and balanced panels and compared our results. The difference in main coefficients was marginal.

3 Using individual fixed effects may introduce a small bias in our regression by limiting our dataset to individual with more than one observation, similarly to a balanced panel. We tested the magnitude of this bias by performing Models 1–4 in Table 4 using unbalanced and balanced panels and compared our results. The difference in main coefficients was only marginal.

## References

[1] Orentlicher D (1996). Paying physicians more to do less: financial incentives to limit care. Univ Richmond Law Rev.

[2] Moffit, R.: A guide to the clinton health plan. Talking Points. The Heritage Foundation

[3] Redmon DP, Yakoboski PJ (1995). The nominal and real effects of hospital global budgets in France. Inquiry.

[4] Bradley C, Fan VY (2015). Strategic provider behavior under global budget payment with price adjustment in Taiwan. Health Econ..

[5] Fan C-P, Chen K-P, Kan K (1998). The design of payment systems for physicians under global budget—an experimental study. J Econ Behav Organ.

[6] Yip WC, Lee Y-C, Tsai S-L, Chen B (2017). Managing health expenditure inflation under a single-payer system: Taiwan’s National Health Insurance. Soc Sci Med.

[7] Benstetter F, Wambach A (2006). The treadmill effect in a fixed budget system. J Health Econ.

[8] Lagasnerie, G.D., Paris, V, Mueller, M., Kumar, A.: Tapering payments in hospitals: experiences in OECD countries. OECD Health Working Papers (2015)

[9] Patel A, Rajkumar R, Colmers JM, Kinzer D, Conway PH, Sharfstein JM (2015). Maryland’s global hospital budgets—preliminary results from an all-payer model. N Engl J Med.

[10] Coulam RF, Gaumer GL (1992). Medicare’s prospective payment system: a critical appraisal. Health Care Financ Rev.

[11] Ellis R, McGuire T (1993). Supply-side and demand-side cost sharing in health care. J Econ Perspect.

[12] Hodgkin D, McGuire TG (1994). Payment levels and hospital response to prospective payment. J Health Econ.

[13] Kroneman M, Boerma W, van den Berg M, Groenewegen P, de Jong J, van Ginneken E (2016). Netherlands: health system review. Health Syst Transit.

[14] Hurley J, Lomas J, Goldsmith LJ (1997). Physician responses to global physician expenditure budgets in canada: a common property perspective. The Milbank Q.

[15] Roberts ET, Hatfield LA, McWilliams JM, Chernew ME, Done N, Gerovich S, Gilstrap L, Mehrotra A (2018). Changes In hospital utilization 3 years into maryland’s global budget program for rural hospitals. Health Aff (Millw).

[16] Detsky AS, Stacey SR, Bombardier C (1983). The effectiveness of a regulatory strategy in containing hospital costs. The Ontario experience, 1967–1981. N Engl J Med.

[17] OECD (2013). Health at a glance 2013: OECD indicators.

[18] van Kleef RC, Eijkenaar F, van Vliet R (2019). Selection incentives for health insurers in the presence of sophisticated risk adjustment. Med Care Res Rev.

[19] Withagen-Koster AA, van Kleef RC, Eijkenaar F (2018). Examining unpriced risk heterogeneity in the Dutch health insurance market. Eur J Health Econ.

[20] Vektis Inzicht in het overstapseizoen. Vektis Intelligence. 5th Edition (2019)

[21] Douven R, Mocking R, Mosca I (2015). The effect of physician remuneration on regional variation in hospital treatments. Int J Health Econ Manag.

[22] Hasaart F (2011). Incentives in the diagnosis treatment combination payment system for specialist medical care.

[23] Beukers PD, Kemp RG, Varkevisser M (2014). Patient hospital choice for hip replacement: empirical evidence from the Netherlands. Eur J Health Econ.

[24] Dutch Hospital Data. Kengetalle nederlandse ziekenhuis 2013. Utrecht, Netherlands (2015). Available: https://www.dhd.nl/klantenservice/paginas/aanvraag-rapport-kostenontwikkeling-ziekenhuiszorg-2013.aspx

