# Supplementary material for: Global budget versus cost ceiling: a natural experiment in hospital payment reform in the Netherlands
Source: Eur J Health Econ. 2019 Sep 16;21(1):105–14. doi: 10.1007/s10198-019-01114-6 (PMC7058687; doi:10.1007/s10198-019-01114-6)
Supplement: Supplementary file 1 — Supplementary material 1 (DOCX 23 kb) [file 10198_2019_1114_MOESM1_ESM.docx]

##### Appendix Table 1: Analysis 1. – Probability of hospital visit – Logistic regressions

| *Dependent variable:* | | | | |  |
| --- | --- | --- | --- | --- | --- |
|  |  | | | | |
|  | treat | | | treat | |
|  | *logistic* | | | *conditional* | |
|  |  | | | *logistic* | |
|  | (1) | (2) | (3) | (4) | (5) |
|  | | | | | |
| Post | -0.032^***^ | 0.057^***^ | -0.012^***^ | -0.025^***^ | -0.036^***^ |
|  | (0.001) | (0.002) | (0.002) | (0.001) | (0.002) |
|  |  |  |  |  |  |
| Post*RCT |  | -0.277^***^ | -0.149^***^ |  | 0.034^***^ |
|  |  | (0.003) | (0.003) |  | (0.006) |
|  |  |  |  |  |  |
| Age Gr.1-4 |  |  | -1.352^***^ |  |  |
|  |  |  | (0.009) |  |  |
|  |  |  |  |  |  |
| Age Gr.5-9 |  |  | -1.497^***^ |  |  |
|  |  |  | (0.009) |  |  |
|  |  |  |  |  |  |
| Age Gr.10-14 |  |  | -1.679^***^ |  |  |
|  |  |  | (0.009) |  |  |
|  |  |  |  |  |  |
| Age Gr.15-19 |  |  | -1.666^***^ |  |  |
|  |  |  | (0.009) |  |  |
|  |  |  |  |  |  |
| Age Gr.20-24 |  |  | -1.639^***^ |  |  |
|  |  |  | (0.009) |  |  |
|  |  |  |  |  |  |
| Age Gr.25-29 |  |  | -1.445^***^ |  |  |
|  |  |  | (0.009) |  |  |
|  |  |  |  |  |  |
| Age Gr.30-34 |  |  | -1.251^***^ |  |  |
|  |  |  | (0.009) |  |  |
|  |  |  |  |  |  |
| Age Gr.35-39 |  |  | -1.294^***^ |  |  |
|  |  |  | (0.009) |  |  |
|  |  |  |  |  |  |
| Age Gr.40-44 |  |  | -1.317^***^ |  |  |
|  |  |  | (0.009) |  |  |
|  |  |  |  |  |  |
| Age Gr.45-49 |  |  | -1.214^***^ |  |  |
|  |  |  | (0.009) |  |  |
|  |  |  |  |  |  |
| Age Gr.50-54 |  |  | -1.021^***^ |  |  |
|  |  |  | (0.009) |  |  |
|  |  |  |  |  |  |
| Age Gr.55-59 |  |  | -0.823^***^ |  |  |
|  |  |  | (0.009) |  |  |
|  |  |  |  |  |  |
| Age Gr.60-64 |  |  | -0.601^***^ |  |  |
|  |  |  | (0.009) |  |  |
|  |  |  |  |  |  |
| Age Gr.65-69 |  |  | -0.331^***^ |  |  |
|  |  |  | (0.009) |  |  |
|  |  |  |  |  |  |
| Age Gr.70-74 |  |  | -0.012 |  |  |
|  |  |  | (0.009) |  |  |
|  |  |  |  |  |  |
| Age Gr.75-79 |  |  | 0.261^***^ |  |  |
|  |  |  | (0.009) |  |  |
|  |  |  |  |  |  |
| Age Gr.80-84 |  |  | 0.307^***^ |  |  |
|  |  |  | (0.009) |  |  |
|  |  |  |  |  |  |
| Age Gr.85+ |  |  | -0.100^***^ |  |  |
|  |  |  | (0.009) |  |  |
|  |  |  |  |  |  |
| SES |  |  | -0.029^***^ |  |  |
|  |  |  | (0.001) |  |  |
|  |  |  |  |  |  |
| Female |  |  | 0.243^***^ |  |  |
|  |  |  | (0.001) |  |  |
|  |  |  |  |  |  |
| Constant | -0.369^***^ | -0.369^***^ | 0.534^***^ |  |  |
|  | (0.001) | (0.001) | (0.008) |  |  |
|  |  |  |  |  |  |
|  | | | | | |
| Observations | 13,638,708 | 13,638,708 | 13,638,708 | 13,638,708 | 13,638,708 |
| Log Likelihood | -9,205,509 | -9,202,064 | -8,660,015 | -2,522,906 | -2,522,891 |
|  | | | | | |
| *Note:* | ^*^p<0.1; ^**^p<0.05; ^***^p<0.01 | | | | |
