# Supplementary material for: Global budget versus cost ceiling: a natural experiment in hospital payment reform in the Netherlands
Source: Eur J Health Econ. 2019 Sep 16;21(1):105–14. doi: 10.1007/s10198-019-01114-6 (PMC7058687; doi:10.1007/s10198-019-01114-6)
Supplement: Supplementary file 2 — Supplementary material 2 (DOCX 23 kb) [file 10198_2019_1114_MOESM2_ESM.docx]

##### Appendix Table 2: Analysis 1. – Probability of hospital visit – Odds ratios

| *Dependent variable:* | | | | |  |
| --- | --- | --- | --- | --- | --- |
|  |  | | | | |
|  | treat | | | treat | |
|  | *logistic* | | | *conditional* | |
|  |  | | | *logistic* | |
|  | (1) | (2) | (3) | (4) | (5) |
|  | | | | | |
| Post | 0.968 | 1.058 | 0.988 | 0.975 | 0.965 |
|  | (1.001) | (1.002) | (1.002) | (1.001) | (1.002) |
|  |  |  |  |  |  |
| Post*RCT |  | 0.758 | 0.862 |  | 1.034 |
|  |  | (1.003) | (1.003) |  | (1.006) |
|  |  |  |  |  |  |
| Age Gr.1-4 |  |  | 0.259 |  |  |
|  |  |  | (1.009) |  |  |
|  |  |  |  |  |  |
| Age Gr.5-9 |  |  | 0.224 |  |  |
|  |  |  | (1.009) |  |  |
|  |  |  |  |  |  |
| Age Gr.10-14 |  |  | 0.187 |  |  |
|  |  |  | (1.009) |  |  |
|  |  |  |  |  |  |
| Age Gr.15-19 |  |  | 0.189 |  |  |
|  |  |  | (1.009) |  |  |
|  |  |  |  |  |  |
| Age Gr.20-24 |  |  | 0.194 |  |  |
|  |  |  | (1.009) |  |  |
|  |  |  |  |  |  |
| Age Gr.25-29 |  |  | 0.236 |  |  |
|  |  |  | (1.009) |  |  |
|  |  |  |  |  |  |
| Age Gr.30-34 |  |  | 0.286 |  |  |
|  |  |  | (1.009) |  |  |
|  |  |  |  |  |  |
| Age Gr.35-39 |  |  | 0.274 |  |  |
|  |  |  | (1.009) |  |  |
|  |  |  |  |  |  |
| Age Gr.40-44 |  |  | 0.268 |  |  |
|  |  |  | (1.009) |  |  |
|  |  |  |  |  |  |
| Age Gr.45-49 |  |  | 0.297 |  |  |
|  |  |  | (1.009) |  |  |
|  |  |  |  |  |  |
| Age Gr.50-54 |  |  | 0.360 |  |  |
|  |  |  | (1.009) |  |  |
|  |  |  |  |  |  |
| Age Gr.55-59 |  |  | 0.439 |  |  |
|  |  |  | (1.009) |  |  |
|  |  |  |  |  |  |
| Age Gr.60-64 |  |  | 0.548 |  |  |
|  |  |  | (1.009) |  |  |
|  |  |  |  |  |  |
| Age Gr.65-69 |  |  | 0.718 |  |  |
|  |  |  | (1.009) |  |  |
|  |  |  |  |  |  |
| Age Gr.70-74 |  |  | 0.988 |  |  |
|  |  |  | (1.009) |  |  |
|  |  |  |  |  |  |
| Age Gr.75-79 |  |  | 1.298 |  |  |
|  |  |  | (1.009) |  |  |
|  |  |  |  |  |  |
| Age Gr.80-84 |  |  | 1.359 |  |  |
|  |  |  | (1.009) |  |  |
|  |  |  |  |  |  |
| Age Gr.85+ |  |  | 0.904 |  |  |
|  |  |  | (1.009) |  |  |
|  |  |  |  |  |  |
| SES |  |  | 0.972 |  |  |
|  |  |  | (1.001) |  |  |
|  |  |  |  |  |  |
| Female |  |  | 1.276 |  |  |
|  |  |  | (1.001) |  |  |
|  |  |  |  |  |  |
| Constant | 0.692 | 0.692 | 1.706^*^ |  |  |
|  |  |  |  |  |  |
|  | | | | | |
| Observations | 13,638,708 | 13,638,708 | 13,638,708 | 13,638,708 | 13,638,708 |
| Log Likelihood | -9,205,509 | -9,202,064 | -8,660,015 | -2,522,906 | -2,522,891 |
|  | | | | | |
| *Note:* | ^*^p<0.1; ^**^p<0.05; ^***^p<0.01 | | | | |
