# Supplementary material for: Global budget versus cost ceiling: a natural experiment in hospital payment reform in the Netherlands
Source: Eur J Health Econ. 2019 Sep 16;21(1):105–14. doi: 10.1007/s10198-019-01114-6 (PMC7058687; doi:10.1007/s10198-019-01114-6)
Supplement: Supplementary file 3 — Supplementary material 3 (DOCX 27 kb) [file 10198_2019_1114_MOESM3_ESM.docx]

| **Appendix Table 3: Analysis 2. – Treatment Intensity** |
| --- |

|  | Dependent variable: log(HCE) | | | | | | |
| --- | --- | --- | --- | --- | --- | --- | --- |
|  | *OLS* | | | | *panel* | | |
|  |  | | | | *linear* | | |
|  | (1) | (2) | (3) | (4) | (5) | (6) | (7) |
|  | | | | | | | |
| Post | 0.079^***^ | 0.113^***^ | 0.098^***^ | 0.065^***^ | 0.033^***^ | 0.043^***^ | 0.034^***^ |
|  | (0.001) | (0.002) | (0.002) | (0.002) | (0.001) | (0.002) | (0.002) |
|  |  |  |  |  |  |  |  |
| Post*RCT |  | -0.110^***^ | -0.096^***^ | -0.042^***^ |  | -0.035^***^ | -0.015^***^ |
|  |  | (0.003) | (0.004) | (0.004) |  | (0.005) | (0.005) |
|  |  |  |  |  |  |  |  |
| UMC |  |  | 0.152^***^ | 0.192^***^ |  |  | 0.230^***^ |
|  |  |  | (0.003) | (0.003) |  |  | (0.005) |
|  |  |  |  |  |  |  |  |
| Age Gr.1-4 |  |  |  | -0.957^***^ |  |  |  |
|  |  |  |  | (0.007) |  |  |  |
|  |  |  |  |  |  |  |  |
| Age Gr.5-9 |  |  |  | -1.194^***^ |  |  |  |
|  |  |  |  | (0.007) |  |  |  |
|  |  |  |  |  |  |  |  |
| Age Gr.10-14 |  |  |  | -1.274^***^ |  |  |  |
|  |  |  |  | (0.007) |  |  |  |
|  |  |  |  |  |  |  |  |
| Age Gr.15-19 |  |  |  | -1.059^***^ |  |  |  |
|  |  |  |  | (0.007) |  |  |  |
|  |  |  |  |  |  |  |  |
| Age Gr.20-24 |  |  |  | -0.963^***^ |  |  |  |
|  |  |  |  | (0.007) |  |  |  |
|  |  |  |  |  |  |  |  |
| Age Gr.25-29 |  |  |  | -0.720^***^ |  |  |  |
|  |  |  |  | (0.007) |  |  |  |
|  |  |  |  |  |  |  |  |
| Age Gr.30-34 |  |  |  | -0.595^***^ |  |  |  |
|  |  |  |  | (0.007) |  |  |  |
|  |  |  |  |  |  |  |  |
| Age Gr.35-39 |  |  |  | -0.669^***^ |  |  |  |
|  |  |  |  | (0.007) |  |  |  |
|  |  |  |  |  |  |  |  |
| Age Gr.40-44 |  |  |  | -0.720^***^ |  |  |  |
|  |  |  |  | (0.007) |  |  |  |
|  |  |  |  |  |  |  |  |
| Age Gr.45-49 |  |  |  | -0.677^***^ |  |  |  |
|  |  |  |  | (0.007) |  |  |  |
|  |  |  |  |  |  |  |  |
| Age Gr.50-54 |  |  |  | -0.604^***^ |  |  |  |
|  |  |  |  | (0.007) |  |  |  |
|  |  |  |  |  |  |  |  |
| Age Gr.55-59 |  |  |  | -0.553^***^ |  |  |  |
|  |  |  |  | (0.007) |  |  |  |
|  |  |  |  |  |  |  |  |
| Age Gr.60-64 |  |  |  | -0.496^***^ |  |  |  |
|  |  |  |  | (0.007) |  |  |  |
|  |  |  |  |  |  |  |  |
| Age Gr.65-69 |  |  |  | -0.408^***^ |  |  |  |
|  |  |  |  | (0.007) |  |  |  |
|  |  |  |  |  |  |  |  |
| Age Gr.70-74 |  |  |  | -0.302^***^ |  |  |  |
|  |  |  |  | (0.007) |  |  |  |
|  |  |  |  |  |  |  |  |
| Age Gr.75-79 |  |  |  | -0.182^***^ |  |  |  |
|  |  |  |  | (0.007) |  |  |  |
|  |  |  |  |  |  |  |  |
| Age Gr.80-84 |  |  |  | -0.137^***^ |  |  |  |
|  |  |  |  | (0.007) |  |  |  |
|  |  |  |  |  |  |  |  |
| Age Gr.85+ |  |  |  | -0.138^***^ |  |  |  |
|  |  |  |  | (0.007) |  |  |  |
|  |  |  |  |  |  |  |  |
| SES |  |  |  | -0.011^***^ |  |  |  |
|  |  |  |  | (0.001) |  |  |  |
|  |  |  |  |  |  |  |  |
| Female |  |  |  | 0.013^***^ |  |  |  |
|  |  |  |  | (0.001) |  |  |  |
|  |  |  |  |  |  |  |  |
| Post*UMC |  |  | 0.104^***^ | 0.079^***^ |  |  | 0.064^***^ |
|  |  |  | (0.005) | (0.005) |  |  | (0.006) |
|  |  |  |  |  |  |  |  |
| Post*RCT*UMC |  |  | -0.017 | 0.103^***^ |  |  | -0.157^***^ |
|  |  |  | (0.012) | (0.012) |  |  | (0.016) |
|  |  |  |  |  |  |  |  |
| Constant | 6.714^***^ | 6.714^***^ | 6.698^***^ | 7.293^***^ |  |  |  |
|  | (0.001) | (0.001) | (0.001) | (0.006) |  |  |  |
|  |  |  |  |  |  |  |  |
|  | | | | | | | |
| Observations | 5,523,626 | 5,523,626 | 5,523,626 | 5,523,626 | 5,523,626 | 5,523,626 | 5,523,626 |
| R^2^ | 0.001 | 0.001 | 0.003 | 0.053 | 0.0003 | 0.0003 | 0.001 |
| Adjusted R^2^ | 0.001 | 0.001 | 0.003 | 0.053 | -0.906 | -0.906 | -0.904 |
|  | | | | | | | |
| *Note:* | ^*^p<0.1; ^**^p<0.05; ^***^p<0.01 | | | | | | |
